# Dietary intakes in people with irritable bowel syndrome

**DOI:** 10.1186/1471-230X-11-9

**Published:** 2011-02-03

**Authors:** Elizabeth A Williams, XuiLi Nai, Bernard M Corfe

**Affiliations:** 1 Human Nutrition Unit, Department of Oncology, University of Sheffield, The Medical School, Beech Hill Road, Sheffield, S10 2RX, UK; 2 Department of Oncology, University of Sheffield, The Medical School, Beech Hill Road, Sheffield, S10 2RX, UK

## Abstract

**Background:**

Irritable Bowel Syndrome (IBS) is a functional bowel disorder characterised by episodes of abdominal pain associated with altered bowel habits. Many IBS sufferers believe that diet may play a role in triggering these episodes and may avoid certain foods. However relatively few studies have undertaken a dietary assessment in IBS sufferers to examine the wider impact of the condition upon diet.

**Methods:**

104 individuals with IBS were recruited and asked to complete a validated food frequency questionnaire (FFQ). The data were analysed against Dietary Reference Values for food energy and nutrients for the United Kingdom and observed intakes for the general population and for differences between IBS subtypes and the UK population.

**Results:**

The data show that the dietary intakes of this population of IBS sufferers met the UK Dietary Reference Values. The average energy intake of the population exceeded the Estimated Average Requirements of the UK population and the balance of macronutrients was favourable. Intakes of selected micronutrients significantly exceeded the reference nutrient intakes. There were no differences between IBS subtypes.

**Conclusions:**

The IBS subpopulation appear to have an adequate and balanced macronutrient intake with no evidence of inadequate micronutrient intake.

## Background

Irritable Bowel Syndrome (IBS) is a functional bowel disorder. It is characterised by episodes of abdominal pain associated with altered bowel habits, in the absence of any structural abnormality or organic lesion. This disorder affects approximately 10% of the world's population [1] In the developed world, IBS is the most commonly diagnosed gastrointestinal disorder and is found predominantly in women[2].

There are three recognised variants of IBS: diarrhoea-predominant (Type D), constipation-predominant (Type C), and an alternating pattern (Type A). The Rome Criteria define a frequency of symptoms including abdominal pain and change in stool frequency, stool consistency or relief of pain upon defecation, bloating, flatulence, passage of mucus, straining, urgency or incomplete evacuation, which allow diagnosis.

Subjects with IBS have lower quality of life, are more likely to use health service resources, and have higher work absenteeism than healthy controls [3]. The cause of IBS remains unknown, although many different etiology theories have been postulated including disrupted gut microbiota function, immunological dysfunction, food allergy/intolerance, altered gut motility, psychological/stress factors and genetic predisposition [4].

Two-thirds of subjects with IBS perceive their symptoms to be diet related [5] and therefore they may restrict their food intake or eliminate certain provocative dietary agents. Reported perceived triggers included carbohydrates and fatty foods, together with caffeine, alcohol and spices. This could potentially distort macronutrient intake and put subjects at risk of low nutrient intakes. To date, few studies have examined the dietary intakes of IBS patients to identify the dietary changes or potential consequent nutrient deficiencies. Of these studies, most used a 3-day dietary record [6,7], or dietary recall[8,9] method to assess the dietary intakes of IBS patients. The food frequency questionnaire (FFQ) has been used less commonly in IBS studies, despite its value in identifying long term or habitual dietary intake [8,10,11].

It is important that the habitual dietary intakes of IBS patients be understood prior to any dietary interventions or recommendations. Herein we aimed to assess the dietary intake of subjects with IBS using a validated FFQ and compared their nutrient intake with that of the UK Dietary Reference Values [12], and population intake averages from the UK National Diet and Nutrition Survey of adults aged 19-64 years,[13,14] primarily hypothesizing that a condition which subjects attributed in part to diet or trigger foods may lead to an unbalanced diet and secondly that symptom management of diarrhoea and constipation via diet may also lead to dietary modification.

## Methods

### Study Participants

Data were collected from 104 participants with IBS prior to an intervention trial with prebiotics, probiotics and synbiotics. Volunteers were recruited via posters and e-mail advertisements in the University of Sheffield, Sheffield Hallam University and on The Gut Trust web-site http://www.theguttrust.org. Inclusion criteria were: aged 18 years and over, and self-reported active IBS as assessed by the Rome II criteria [15]. Exclusion criteria were a history of gastrointestinal surgery, pregnant or lactating, diabetic, were supplementing their habitual diet with prebiotics, probiotics and synbiotic products, or were taking antibiotics or medication for their IBS. Written, informed consent was obtained from all participants. Ethical approval for this study was granted by The University of Sheffield Research Ethics Committee (SMBRER83). Participant weight and height was self-reported. Participants were also asked to complete a validated questionnaire to assess current IBS symptoms [16]. This scores subjects as having no symptoms (< 75), mild (75-175), moderate (175-300) or severe IBS (> 300).

### Dietary assessment

Volunteers were asked to complete a self-administered food frequency questionnaire (FFQ) which was a modified version of the EPIC FFQ which has been previously validated in a population of UK adults [17]. This is a retrospective method of dietary assessment that has been designed to assess total dietary intake. The questionnaire asked participants to indicate the frequency of consumption of 134 food types over the previous 12 months. Participants had nine choices to indicate the frequency of consumption of each of the 134 foods from 'never or less than once a month' to '6+ times per day'. Foods in the questionnaire are grouped into categories: meat & fish, bread and savoury biscuits, cereals, potatoes, rice and pasta, dairy products & fats, sweets and snacks, soups, sauces and spreads, drinks, fruits, and vegetables. The questionnaire also includes additional questions on milk consumption, fats used and cooking methods. The modifications made to the original EPIC questionnaire were minor and included the addition of some frequently consumed foods e.g. Yorkshire pudding, garlic bread and the removal of food items that were known to be infrequently consumed by the population e.g. cods roe. FFQ data were entered into SPSS and analysed using an in-house database, using a similar approach to that described previously whereby the FFQ database is linked to a nutrient database to allow total nutrient intake to be estimated for each participant [18]. The analysis was based on the 5^th ^edition of The Composition of Foods [19] and in-house portion size data derived from a dietary survey of UK adults [20].

To examine the energy and nutrient adequacy of the diet the self-reported dietary intakes were compared to the relevant UK Dietary Reference Values for adults aged 19-50 years [12] thereby allowing us to identify if, as a population, the participants were meeting current UK recommended requirements.

The mean daily intakes were also compared with mean values obtained from the UK National Diet and Nutrition Survey [13,14]. This nationally representative survey assessed the diet of >1700 adults aged 19-64 years using seven day weighed intakes. The survey took place in 2000/2001, and is the most recent and best available national dietary intake data available on UK adults. This survey presents dietary intakes according to gender. Comparison of the dietary intake of participants in this study were therefore compared with the survey data according to gender.

### Statistical analysis

All data were analysed using SPSS (SPSS Inc, Chicago, IL, USA). Comparisons of the effect of gender were undertaken using an unpaired t-test; comparisons between IBS subtypes' were undertaken using one-way Analysis of Variance. Two tailed one sample t-tests were used to compare intakes with Dietary Reference Values and with the UK National Diet and Nutrition Survey. P-values less than 0.05 were considered statistically significant.

## Results

### Basic demographics

Participant characteristics are shown in Table 1. In total, 104 subjects with IBS participated in the study and completed the food frequency questionnaire, of whom 22% (23) were male and 78% (81) were female. Forty percent of the participants reported alternating IBS symptoms (Type A), 38% diarrhoea predominant (Type D) and the remaining 22% reported constipation (Type C) predominant symptoms. The distribution of the IBS subtypes was similar in female and male subjects (data not shown).

**Table 1:** Characteristics of the 104 participants by IBS subtype and gender

	All participants n = 104	IBS subtype†			Gender‡	
		
		Type A n = 42	Type C n = 23	Type D n = 39	Men n = 23	Women n = 81
**Age, years *(mean ± SD)***	33.3 ± 13.90	32.1 ± 12.97	33.4 ± 12.79	34.6 ± 15.63	43.3 ± 15.49	30.5 ± 12.08*

**BMI, kgm^-2 ^*(mean ± SD)***	24.1 ± 4.48	23.0 ± 3.75	24.1 ± 3.81	25.3 ± 5.30	25.7 ± 4.32	23.7 ± 4.45

**Total symptom severity** ***(mean ± SD)***	250 ± 75.7	256 ± 63.0	244 ± 83.9	250 ± 83.9	223 ± 74.7	258 ± 74.8

† Differences between IBS subtypes analysed by one way analysis of variance. There was no significant difference in age, BMI or total symptom severity between the 3 IBS subtypes (A-alternating, C- constipation, D- diarrhoea).

‡ Differences between genders analysed by unpaired students t-test. * P < 0.05

The mean age of the volunteers was 33 years, with females participants (31 years old) significantly younger than male participants (43 years old) (t = 3.674, P < 0.01). There was no significant difference in mean age across the IBS subtypes. Male participants had a mean Body Mass Index (BMI) of 25.7 kg/m^2^, and were not significantly different from a comparable age group in the National Diet and Nutrition Survey where the mean BMI was 27.2 kg/m^2^. Female participants had a mean BMI of 23.7 kg/m^2^, significantly lower than their National Diet and Nutrition Survey counterparts who had a mean BMI of 26.4 kg/m^2 ^(t = -5.327, P < 0.001). The majority of this population had a self-reported BMI within the normal range (BMI 18.5-25), however 5.2% of women were underweight (BMI < 18.5), and 45.4% of men and 28.6% of women were overweight or obese (BMI >25). In the National Diet and Nutrition Survey 1% of men and 3% of women were underweight and 66% of men and 53% of women overweight or obese. Participants reported a mean IBS total symptom severity score of 250, indicative of moderate IBS. There was no significant difference in the reported symptom severity according to IBS subtype or gender.

### Dietary intake of IBS participants compared with dietary recommendations

The mean (±SD) daily intakes for energy, macronutrients and selected micronutrients for participants are presented in Table 2 and are compared against Dietary Reference Values, where available [12]. Energy intake was significantly higher than the Estimated Average Requirements (P = 0.01). Protein intake was significantly higher than the Reference Nutrient Intake for protein (P < 0.001) however when this was expressed as the percentage energy from diet, intake did not differ significantly from the population average Dietary Reference Values. Reference nutrient intakes are not available for absolute intakes of fat and carbohydrate however percent energy from fat was significantly lower (P < 0.001) and percent energy from carbohydrate significantly higher (P < 0.001) than Dietary Reference Values. Daily intake of non-starch polysaccharides significantly exceeded the recommended target intake of 18 g/day and all micronutrients analysed significantly exceeded Reference Nutrient Intakes.

**Table 2:** Daily energy and nutrient intake of the study population (mean ± SD) compared with Dietary Reference Values (where available)

Total daily intakes	IBS participants (n = 104)	DRV	***P***^‡^
**Energy intake (MJ)**	9.74 ± 3.163	8.652^1^	0.01

**Carbohydrate (g)**	298 ± 100.5	-	-

**Protein (g)**	86.5 ± 30.89	47.3^2^	< 0.001

**Fat (g)**	79.3 ± 33.27	-	-

**% energy from carbohydrates**	51.4 ± 7.15	47^3^	< 0.001

**% energy from protein**	14.9 ± 2.65	15^3^	0.783

**% energy from fat**	29.8 ± 5.41	33^3^	< 0.001

**% energy from alcohol**	5.5 ± 6.90	-	-

**Non-starch polysaccharides (g)**	23.4 ± 9.39	18^3^	< 0.001

**Calcium (mg)**	1012 ± 381.4	700^4^	< 0.001

**Vitamin C (mg)**	158 ± 67.5	40^4^	< 0.001

**Folate (μg)**	354 ± 111.0	200^4^	< 0.001

**Riboflavin (mg)**	2.0 ± 0.73	1.14^5^	< 0.001

DRV: UK Dietary Reference Values (Department of Health 1991)

^1^Estimated average requirement for energy intake, weighted for the study population. Estimated average requirements for energy intake for men and women aged 19-50 years are 10.6 MJ/day and 8.10 MJ/day respectively.

^2 ^Reference Nutrient Intake for protein, weighted for the study population. Reference nutrient intakes for protein for men and women aged 19-50 years are 55.5 mg/day and 45 mg/day respectively.

^3^Population average

^4 ^Reference Nutrient Intake

^5 ^Reference Nutrient Intake for riboflavin, weighted for the study population. Reference nutrient intakes for riboflavin for men and women aged 19-50 years are 1.3 mg/day and 1.1 mg/day respectively.

***^‡^***Significance was assessed using two-tailed one-sample t-tests.

### Dietary intake of IBS participants compared to a National Dietary Intakes

The mean daily intake (±SD) for energy, macronutrients and selected micronutrients for men and women with IBS and the mean (±SD) daily intakes for men and women as reported in the UK National Diet and Nutrition Survey are presented in Table 3.

**Table 3:** Daily energy and nutrient intake of the study population (mean ± SD) by gender compared to the National Diet and Nutrition Survey

Total daily intakes	IBS Men (n = 23)	NDNS Men†	***P***^‡^	IBS Women (n = 81)	NDNS Women†	***P***^‡^
**Energy intake (MJ)**	10.86 ± 3.435	8.88 ± 2.388	0.011	9.422 ± 3.027	6.54 ± 1.730	< 0.001
						
**Carbohydrate (g)**	335 ± 115.0	275 ± 79	0.020	287 ± 94.0	203 ± 59	< 0.001
**Protein (g)**	92.0 ± 31.64	88.2 ± 32.67	0.574	84.9 ± 30.69	63.7 ± 16.61	< 0.001
**Fat (g)**	90.2 ± 36.84	86.5 ± 28.17	0.636	76.1 ± 31.72	61.4 ± 21.72	< 0.001
**% energy from carbohydrates**	52.1 ± 8.11	47.7 ± 6.03	0.017	51.2 ± 6.90	48.5 ± 6.72	0.001
**% energy from protein**	14.2 ± 2.41	16.5 ± 3.63	< 0.001	15.1 ± 2.69	16.6 ± 3.50	< 0.001
**% energy from fat**	30.3 ± 5.15	35.8 ± 5.63	< 0.001	29.6 ± 5.50	34.9 ± 6.52	< 0.001
						
**% energy from alcohol**	6.3 ± 7.17	6.5 ± 7.20	0.886	5.2 ± 6.85	3.9 ±5.11	0.087
**Non-starch polysaccharides (g)**	25.9 ± 10.12	15.2 ± 6.04	< 0.001	22.7 ± 9.09	12.6 ± 5.01	< 0.001
**Calcium (mg)**	1046 ± 352.9	1007 ± 411.2	0.604	1002 ± 390.8	777 ± 268.7	< 0.001
**Vitamin C (mg)**	177 ± 80.0	83.4 ± 54.45	< 0.001	152 ± 62.9	81.0 ± 49.93	< 0.001
						
**Folate (μg)**	412 ± 121.4	344 ± 126.8	0.014	337 ± 102.8	251 ± 89.9	< 0.001
**Riboflavin (mg)**	2.2 ± 0.75	2.11 ± 0.939	0.626	2.0 ± 0.72	1.6 ± 0.638	< 0.001

† Mean daily intakes from food sources only of men and women in the UK National Diet and Nutrition Survey (Henderson et al. 2003)

*^‡^*Significance was assessed using two-tailed one-sample t-tests.

The mean self-reported energy intake for both men and women was significantly higher than reported in the National Diet and Nutrition Survey. Carbohydrate intake and the percent energy derived from carbohydrate were significantly higher in both genders compared with the National Diet and Nutrition Survey data. Absolute protein intake was significantly higher in IBS women compared with the National Diet and Nutrition Survey, no significant difference was observed in men; however percent energy derived from protein was significantly higher in both genders than reported in the National Diet and Nutrition Survey. Absolute fat intake was significantly higher in IBS women compared with the National Diet and Nutrition Survey, no significant difference was observed in the men; however when fat intake was expressed as percent energy both men and women reported significantly less energy derived from fat than in the National Diet and Nutrition Survey. Percent energy derived from alcohol was not significantly different from the National Diet and Nutrition Survey in either gender. The selected micronutrients, calcium, vitamin C, folate and riboflavin were all significantly higher in female participants than in the National Diet and Nutrition Survey. For males, significantly higher micronutrient intakes were only reported for vitamin C and folate.

### Dietary intake of participants according to IBS subtype

Finally, we sought to establish whether there was any difference between IBS subtypes. Table 4 summarizes the mean (±SD) daily intake of energy, macronutrients and selected micronutrients of participants according to IBS subtype. No significant difference was observed for intake of energy and nutrients between the IBS subtypes.

**Table 4:** Daily energy and nutrient intake of the study population (mean ± SD) by IBS subtype

Total daily intakes	Type A (n = 42)	Type C (n = 23)	Type D (n = 39)
**Energy intake (MJ)**	9.228 ±3.264	10.204 ± 3.826	10.036 ± 2.560
			
**Carbohydrate (g)**	283 ± 94.6	316 ± 129.5	303 ± 88
**Protein (g)**	79.0 ± 28.36	91.4 ± 31.44	91.8 ± 32.28
**Fat (g)**	76.1 ± 36.23	82.6 ± 42.52	80.8 ± 23.12
**% energy from carbohydrates**	51.8 ± 7.57	52.1 ± 6.91	50.7 ±6.94
**% energy from protein**	14.5 ± 2.29	15.2 ± 2.52	15.3 ± 3.05
**% energy from fat**	30.1 ± 6.42	28.9 ± 5.69	29.8 ± 3.93
			
**% energy from alcohol**	5.5 ± 7.07	5.4 ± 6.80	5.5 ± 6.95
**Non-starch polysaccharides (g)**	22.3 ± 8.05	25.1 ± 9.52	23.7 ± 10.66
			
**Calcium (mg)**	961 ± 382	1078 ± 407.8	1030 ± 368.0
**Vitamin C (mg)**	154 ± 67.0	159 ± 57.9	161 ± 74.2
**Folate (μg)**	339 ± 111.9	381 ± 127.8	355 ± 99.5
			
**Riboflavin (mg)**	1.9 ± 0.65	2.1 ± 0.88	2.1 ± 0.72

Significance was assessed using one way analysis of variance. There was no significant difference in dietary intake between the 3 IBS subtypes.

## Discussion

The purpose of our study was to assess the dietary intakes of subjects with IBS and whether any alteration in dietary pattern had impacted on nutrient intake. The participants in this study reported consuming a diet with a macronutrient composition that met recommended requirements. The micronutrient intakes that were measured all exceeded Reference Nutrient Intakes. This suggests that participants' alteration or restriction of their diet does not adversely affect nutrient intake.

Body Mass Index is the most commonly used method to identify malnourished adults. Compared with the UK National Diet and Nutrition Survey females with IBS have a significantly lower BMI. A higher proportion of the IBS population were classified as underweight, and a smaller proportion were overweight or obese. The propensity for a lower BMI in the IBS population particularly among women may reflect that the BMI was calculated from self-reported weight and height and individuals have a tendency to under-report their weight. However, despite a lower average BMI the estimated average energy intake of the population was higher than the National Diet and Nutrition Survey data, this apparent discrepancy may reflect increased energy intake requirements due to abnormal bowel motility and malabsorption, or may reflect increased energy expenditure due to increased metabolic rate or higher levels of physical activity. Further research is needed to help explain this anomaly.

Whilst some previous studies have suggested nutritional inadequacies in IBS populations, this study did not. Possible reasons for this include biogeographical aspects (few previous studies have addressed the UK population) and limitations of the FFQ by comparison with food diaries for estimation of dietary intake. It must also be recognised that subjects were recruited from the general population and at the time of study were managing symptoms independently. Such a population are likely to differ from subjects recruited via gastroenterology clinics or subjects requiring medication where the impact of this disorder on the nutritional intake and status may be more acute.

The Reference Nutrient Intake of a vitamin or mineral is the quantity of that micronutrient that satisfies the requirements of 97% of the population. In this study average intakes significantly exceeded the Reference Nutrient Intakes for all micronutrients considered, suggesting that there is a low risk of deficiency in the population. No significant difference was found between the IBS subtypes for energy and other nutrients. We did nevertheless note that our population had a higher percentage of energy coming from carbohydrate and a lower percentage coming from fat than reported in the National Diet and Nutrition Survey. There were also higher than average intakes of non-starch polysaccharides, folate and vitamin C. This may reflect misreporting of certain nutrient rich foods such as fruit and vegetable intake. These trends may be indicative of a population especially receptive to a healthy eating propaganda or motivated to compensate for restriction of trigger foods. Nonetheless this may be an area for more detailed future investigation to ensure it does not mask deficiencies not tested in this analysis and suggested by other studies [9,10].

Caution should be exercised in the interpretation of intake data using FFQs. This particular FFQ has been reported to overestimate the intake of fruit and vegetables and non-starch polysaccharides [21,22] and this is probably reflected in the higher than expected vitamin C and non-starch polysaccharides. Nevertheless the FFQ is believed to perform well for the overall assessment of macronutrient contribution to the total energy intake [20]. In two FFQ-based surveys one found no change in energy or macronutrient intake compared with control groups [10], whilst a second identified a reduction in carbohydrate and increase in fat intake [8]. Nevertheless, in our study, intakes of macronutrients and those micronutrients analysed were all above Dietary Reference Values. Whilst the FFQ used in the present study tends to overestimate dietary intakes [23], the tool has the benefit of estimating more habitual patterns of intake. Similar intakes of vitamin C, calcium and non-starch polysaccharides were reported previously [22] using the same FFQ assessment methods. Furthermore all nutrients tended to follow the same trend of slightly elevated values against Dietary Reference Values, with no intakes being obviously different, again providing confidence in the conclusion.

There are limitations of dietary assessment using Food Frequency Questionnaire that must be acknowledged. The FFQ used asks participants to estimate their dietary intake over the previous 12 months. As with all retrospective methods the intakes may therefore be subject to recall bias. A prospective method of dietary assessment such as a food diary could be used, however the presence of active IBS symptoms may distort dietary intake and symptom severity would need to be taken into account.

We chose to compare the dietary intakes of the IBS participants with the intakes reported in the UK National Diet and Nutrition Survey. This survey provides the most recent information on the dietary intakes of a representative sample of the UK population. The National Diet and Nutrition Survey used a 7-day weighed food diary. The differences in the IBS intakes compared with the National Diet and Nutrition Survey may reflect the different dietary assessment methods. All dietary assessment methodologies are subject to measurement error and food diaries are prone to under-reporting. A combination of over-estimation using a FFQ and an underestimation using a food diary may in part account for the differences observed. Nevertheless there was no suggestion that participants had inadequate dietary intake and contrary to expectations there was a suggestion that intakes were better than the general population as indicated by a lower % energy derived from fat and more optimal BMI.

Further analysis of the diet is needed to identify whether specific food items that are believed to exacerbate symptoms are avoided. However this study suggests that if specific foods are eliminated then there is successful substitution and manipulation of the diet with other foods rich in the key nutrients in this population.

## Conclusions

We have used a dietary assessment tool aimed at estimating habitual dietary intake to examine the diet of 104 people with IBS. There are few reported studies which have used this strategy, particularly in the UK population. Our data suggest that the intakes in this population are generally adequate irrespective of IBS subgroup. Any individual foods excluded from the diet of this population have not resulted in a detectable deficiency of intake.

## Competing interests

The study was funded by Obsidian Ltd.

## Authors' contributions

XLN undertook the analysis of data and drafted the first version of the manuscript, EAW supervised the collection and analysis of data, and undertook the analysis of FFQ and edited the manuscript, BMC conceived and directed the study, supervised the collection and analysis of data and edited the manuscript.

## Pre-publication history

The pre-publication history for this paper can be accessed here:

http://www.biomedcentral.com/1471-230X/11/9/prepub
